# PD119 Interventions To Improve Long COVID Symptoms: A Systematic Review Of Randomized Controlled Trials

**DOI:** 10.1017/S0266462324003593

**Published:** 2025-01-07

**Authors:** Cillian McDowell, Barrie Tyner, Shibu Shrestha, Leah McManus, Fearghal Comaskey, Patricia Harrington, Kieran A. Walsh, Michelle O’ Neill, Máirín Ryan

## Abstract

**Introduction:**

Long COVID, which encompasses a range of prolonged and persistent symptoms that occur after the acute SARS-CoV-2 infection period, can have substantial negative physical, mental, social, and economic effects. This systematic review aimed to assess the effectiveness and safety of interventions to improve long COVID symptoms to inform updates to the interim long COVID model of care in Ireland.

**Methods:**

Studies were identified in the MEDLINE, Embase, and CENTRAL databases through February 2023. Inclusion criteria were: (i) participants with long COVID, as defined by the study authors; (ii) random assignment to either an intervention or a comparison group; and (iii) quantitative assessment of the severity or frequency of long COVID symptoms. Exclusion criteria were: (i) signs or symptoms not reasonably attributable to prior SARS-CoV-2 infection; (ii) interventions not intended to treat long COVID; and (iii) not a randomized controlled trial. Two reviewers independently screened studies, extracted data, and assessed study quality using the Cochrane risk-of-bias tool for randomized trials. The results were synthesized narratively.

**Results:**

Fifty-seven studies were included, and 283 potentially relevant ongoing trials were identified. Twenty-four trials investigated pharmaceutical and other medical interventions, most of which were examined in single studies. Thirty-three trials investigated non-pharmaceutical interventions. Risk of bias was high in 41 of the 57 (72%) studies. Interventions targeted a diverse range of long COVID symptoms. Studies generally had small sample sizes and short follow-up periods and did not adequately examine intervention safety. Evidence for the effectiveness of pharmaceutical and other medical interventions was limited. Potential short-term improvements were seen for some people following personalized exercise and physiotherapy and rehabilitation programs. However, long-term outcomes were not assessed.

**Conclusions:**

Effective interventions to improve the symptoms of long COVID remain elusive and those included in this review do not yet have sufficient evidence to support them. In the absence of strong evidence for specific interventions, a holistic approach should be used to support people with long COVID.

